# Enhanced ocean CO_2_ uptake due to near-surface temperature gradients

**DOI:** 10.1038/s41561-024-01570-7

**Published:** 2024-10-25

**Authors:** Daniel J. Ford, Jamie D. Shutler, Javier Blanco-Sacristán, Sophie Corrigan, Thomas G. Bell, Mingxi Yang, Vassilis Kitidis, Philip D. Nightingale, Ian Brown, Werenfrid Wimmer, David K. Woolf, Tânia Casal, Craig Donlon, Gavin H. Tilstone, Ian Ashton

**Affiliations:** 1 Faculty of Environment, Science and Economy, University of Exeter, Penryn, UK; 2 Plymouth Marine Laboratory (PML), Plymouth, UK; 3 Ocean and Earth Science, University of Southampton, Southampton, UK; 4 School of Energy, Geoscience, Infrastructure and Society, Heriot Watt University, Stromness, UK; 5 European Space Agency (ESA-ESTEC), Noordwijk, The Netherlands

**Keywords:** Marine chemistry, Carbon cycle

## Abstract

The ocean annually absorbs about a quarter of all anthropogenic carbon dioxide (CO_2_) emissions. Global estimates of air–sea CO_2_ fluxes are typically based on bulk measurements of CO_2_ in air and seawater and neglect the effects of vertical temperature gradients near the ocean surface. Theoretical and laboratory observations indicate that these gradients alter air–sea CO_2_ fluxes, because the air–sea CO_2_ concentration difference is highly temperature sensitive. However, in situ field evidence supporting their effect is so far lacking. Here we present independent direct air–sea CO_2_ fluxes alongside indirect bulk fluxes collected along repeat transects in the Atlantic Ocean (50° N to 50° S) in 2018 and 2019. We find that accounting for vertical temperature gradients reduces the difference between direct and indirect fluxes from 0.19 mmol m^−2^ d^−1^ to 0.08 mmol m^−2^ d^−1^ (*N* = 148). This implies an increase in the Atlantic CO_2_ sink of ~0.03 PgC yr^−1^ (~7% of the Atlantic Ocean sink). These field results validate theoretical, modelling and observational-based efforts, all of which predicted that accounting for near-surface temperature gradients would increase estimates of global ocean CO_2_ uptake. Accounting for this increased ocean uptake will probably require some revision to how global carbon budgets are quantified.

## Abstract (web summary)

Accounting for near-surface temperature gradients leads to estimates for annual CO_2_ uptake in the North Atlantic that are 7% higher, based on a comparison of eddy covariance and bulk CO_2_ measurements, which is consistent with theory, laboratory assessments and model analysis.

## Main

The oceans form a critical component of the global carbon cycle and represent a long-term net sink of anthropogenic carbon dioxide (CO_2_)^1^. In 2021, CO_2_ uptake by the oceans was quantified as 2.9 ± 0.4 PgC yr^−1^ (ref. ^2^), which equates to ~25% of the anthropogenic CO_2_ emissions^2^. Estimates of the oceanic carbon sink (derived from measurements of CO_2_ in bulk air and seawater) provide one of two critical observational constraints on the global carbon budget^3,4^; the other being atmospheric observations. Therefore, advances in our understanding of the processes that control air–sea CO_2_ exchange and resulting net transfer improve the closure of the global carbon budget and any resulting policy advice^5^.

**Fig. 1:**
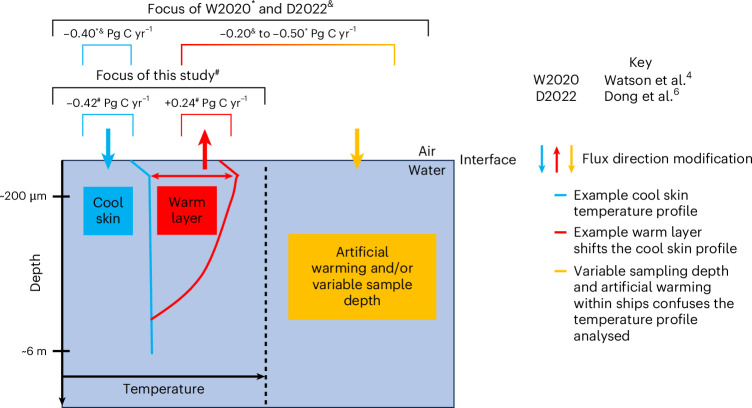
Schematic indicating the modulation of air–sea CO_2_ fluxes by vertical temperature gradients. Example natural temperature profiles indicate a well-mixed profile with the cool skin and a profile with an exemplar warm layer. The artificial warming and variable sampling depth of ships that can confuse the temperature (and concurrent CO_2_) profiles is presented. Arrows indicate the direction of the air–sea CO_2_ flux modifications from a profile with no vertical temperature gradients. Coloured brackets indicate the different components focused on within the studies listed in the key and the estimated global net impact of each component as described within the text. Superscript symbols link the studies to their respective global correction values above the brackets for the vertical temperature gradients covered. Data from refs. ^4,6^.

Previous studies have identified that the ocean carbon sink estimated from bulk air and seawater CO_2_ may be underestimated due to overlooking naturally occurring vertical temperature gradients that are known to exist in the water close to the ocean’s surface^3,4,6–10^. There are two important natural effects. First, the temperature at the air–sea interface (the top of the water mass boundary layer, practically approximated as the skin temperature; *T*_skin_, ≈10 μm depth) is known to be ubiquitously cooler than the water below (that is, the bottom of the mass boundary layer or subskin at ≈2 mm depth). This characteristic is known as the cool skin effect^11–13^ and is caused by heat leaving the water as it is in direct contact with the atmosphere. Second, over greater water depths and under conditions of high insolation and low wind speed, the top few metres of the ocean are heated relative to the waters further below, which is known as the diurnal warm layer. But even here the cool skin exists due to the persistant heat fluxes at the air–water interface. Recent theoretical evaluations indicate that the cool skin and warm layers have opposing effects on the air–sea CO_2_ flux, whereby the cool skin increases ocean uptake and the warm layers can decrease uptake^9^ (Fig. 1). Consequently, estimating the ocean CO_2_ sink using measurements of seawater CO_2_ and temperature from a typical ship’s intake depth (*T*_depth_) of ~5–8 m and atmospheric CO_2_ at a height of ~20 m necessitates a careful assessment of what is happening near the air–water interface, where the actual CO_2_ exchange takes place.

Our knowledge of the impact of these natural near-surface vertical temperature gradients on gas concentrations and air–sea gas fluxes comes from multiple sources. The existence of the cool skin and diurnal warming temperature gradients is well established within the sea surface temperature communities, prompting the need for depth specific temperature nomenclature and datasets (given by the internationally accepted Global High Resolution Sea Surface Temperature, GHRSST definitions, for example, as outlined by Donlon et al.^12^ and Merchant et al.^14^). Within the carbon community, early work by Robertson and Watson^8^ postulated the potential impact of the cool skin effect on air–sea CO_2_ exchange. The existence of near-surface gas concentration gradients for multiple gases and their link to temperature gradients were proposed^15^ and then imaged in a wave tank for poorly soluble oxygen^16^. Subsequently, the importance of temperature gradients and their influence on CO_2_ gas concentrations and air–sea exchange of CO_2_ has been collated and reviewed^9^ and concluded that overlooking these influences can result in a bias in air–sea CO_2_ flux. Correcting for temperature gradients to remove this bias is achieved by adjusting the CO_2_ concentrations according to the observed or expected changes in temperature across the mass boundary layer. Recent observation-based work has predicted that these natural corrections (cool skin and warm layers), with the addition of an artificial component due to warming of samples within the ship’s intake before measurement, could increase the global ocean CO_2_ sink by between 0.3 to 0.9 PgC yr^−1^, or between 10 and 31% of the global ocean sink (of 2.9 PgC yr^−1^)^3,4,6,9^ (Fig. 1). Although the understanding of the possible impact of these vertical temperature gradients on air–sea gas fluxes is growing, their inclusion is still missing from most indirect air–sea gas flux estimates and assessments^2,17^. The slow exploitation of these advancements is due to the lack of in situ field evidence to date^2^, which would provide a missing piece of evidence to help confirm their significance.

**Fig. 2:**
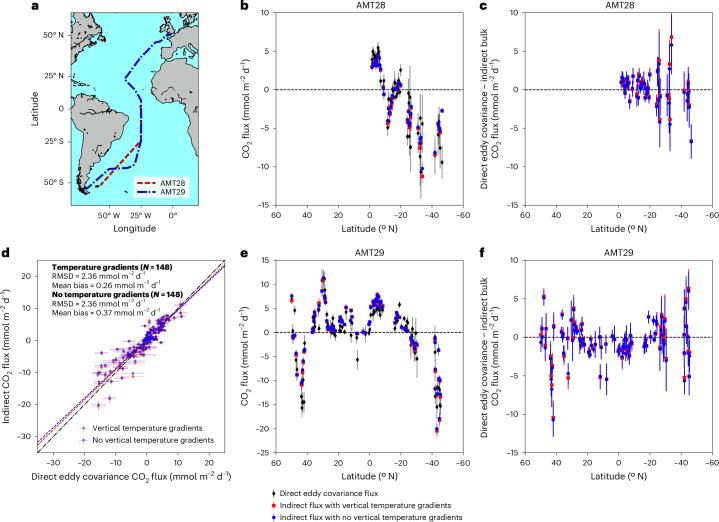
AMT28 and AMT29. **a**, Map of cruise tracks for AMT28 and AMT29. **b**, AMT28 air–sea CO_2_ flux directly measured by the eddy covariance system and indirect CO_2_ fluxes not accounting for and accounting for vertical temperature gradients using the Donlon et al.^12^ cool skin. Dashed line indicates 0. **c**, AMT28 difference between direct eddy covariance air–sea CO_2_ flux and indirect bulk fluxes not accounting for vertical temperature gradients (blue circles) and accounting for vertical temperature gradients using the Donlon et al.^12^ cool skin (red circles). Dashed line indicates 0. **d**, Comparison between 3-h mean direct eddy covariance and indirect CO_2_ fluxes not accounting and accounting for vertical temperature gradients using Donlon et al.^12^ skin. Dashed lines indicate the Type II linear regression fit for the respective dataset. Dashed-dotted line is the 1:1. In-plot statistics are root mean square difference (RMSD), mean bias and number of 3-h mean samples (*N*). **e**, Same as **b** but for AMT29. Note different *y*-axis limits compared to **b**. **f**, Same as **c** but for AMT29. Error bars in **b**–**f** indicate the calculated uncertainty on the 3-h mean. Basemap in **a** from Natural Earth v4.0.0 (https://www.naturalearthdata.com/).

In this paper, we present in situ observations from two Atlantic Meridional Transect (AMT) cruises, which sampled the South Atlantic Ocean (0° N to 50° S) in 2018 (AMT28) and both the North and South Atlantic oceans (50° N to 50° S) in 2019 (AMT29) (Fig. 2a). Observations included direct CO_2_ flux measurements by the eddy covariance method and indirect bulk CO_2_ fluxes estimated from air and seawater CO_2_ measurements. In situ *T*_skin_ and *T*_depth_ are used to characterize the natural vertical temperature gradients and to evaluate the effect and significance of the cool skin^11–13,18^ and warm layers^9^ on the CO_2_ flux. These independent estimates enable a quantitative assessment of the importance of vertical temperature gradients. Our results provide ocean-scale in situ experimental evidence to support the theoretical and laboratory understanding of near-surface vertical temperature gradients and their impact on air–sea CO_2_ fluxes. Furthermore, the in situ methods presented, and resultant high-quality data produced, could form the basis for creating a fiducial reference dataset for assessing global observational-based data products, which is needed for supporting global carbon observing capabilities^19^.

## Comparison of direct and indirect CO_2_ fluxes

The direct (eddy covariance) and indirect (bulk) CO_2_ flux observations used in this study are state-of-the-art measurements that have well-defined uncertainties and are calibrated to reference standards (Methods). The direct and indirect CO_2_ fluxes showed similar spatial variability throughout the cruise tracks (Fig. 2b,e). For both research cruises, which occurred in boreal autumn (austral spring), the high latitudes acted as CO_2_ sinks, whereas the subtropics and equatorial regions fluctuated between source and sink (Fig. 2b,e). This spatial pattern is largely consistent with previous basin-wide flux estimates^17,20–22^. There was interannual variability in the air–sea CO_2_ flux between the two cruises in the South Atlantic (Fig. 2b,e), where AMT29 showed a weaker CO_2_ sink (Fig. 2e). This feature of higher interannual variability in the partial pressure of CO_2_ (*p*CO_2 (sw)_) during austral spring has been previously identified^23^ and it alters the air–sea CO_2_ flux on interannual timescales^24^.

The direct eddy covariance CO_2_ fluxes implicitly contain the impacts of the competing natural temperature gradient processes that control the air–sea CO_2_ flux, whereas indirect bulk fluxes are based on relevant data combined with simplified models and require the explicit inclusion or exclusion of vertical temperature gradients within the calculations (Methods). When the measured vertical temperature gradients were explicitly included in the indirect fluxes, the mean bias between the direct and indirect fluxes was smaller, indicating improved accuracy and agreement (Fig. 2c,d,f and Table 1), whereas the root mean squared difference remained unchanged. The bias reduced from 0.19 mmol m^−2 ^d^−1^, when the vertical temperature gradients were not accounted for within the bulk flux estimates, to 0.08 mmol m^−2^ d^−1^ (*N* = 148) (Table 1 and Fig. 2d). These results remained consistent when using all commonly used and recent gas transfer parameterizations^25–28^ (Supplementary Tables 1–3) and multiple methods to parameterize the cool skin effect^11–13,18^ (Table 1 and Supplementary Tables 1–3). The temperature corrections resulted in CO_2_ sink regions becoming stronger and CO_2_ sources becoming weaker (unless the warm layer correction is greater than the cool skin correction), which is consistent with the theory^9^. Hereafter, we focus our further discussion on the most commonly used gas transfer parameterization of Wanninkhof et al.^28^, along with the Donlon et al.^12^ cool skin. The latter is the most complex cool skin approach that can be driven by the in situ data collected, whereas the COARE 3.5 approach requires additional model re-analysis for incoming and outgoing radiation data, which may not precisely represent the true local conditions. The equivalent results for all gas exchange parameterizations and cool skin approaches analysed can be found in Supplementary Tables 1–3.

**Table 1:** Statistical comparisons between direct and indirect CO_2_ fluxes not accounting for vertical temperature gradients and accounting for vertical temperature gradients using different cool skin estimates

Method		Mean bias (mmol m^−2^ d^−1^)	RMSD (mmol m^−2^ d^−1^)	Slope	Intercept	*N*
No VTG (equation (2))		0.37	2.36	0.92	0.33	148
Cool skin correction (equation (3))	Fixed skin (0.17 K)	0.11	2.33	0.92	0.07	148
	Donlon et al.^12^ skin	0.11	2.33	0.92	0.07	148
	COARE skin	0.05	2.33	0.92	0.01	148
Cool skin and warm layer correction (equation (4))	Fixed skin (0.17 K)	0.26	2.36	0.94	0.23	148
	Donlon et al.^12^ skin	0.26	2.36	0.94	0.23	148
	COARE skin	0.30	2.36	0.94	0.26	148

Statistical acronyms are mean bias, root mean square difference (RMSD) and number of 3-h mean samples (*N*). VTG, vertical temperature gradients.

## Impacts at different wind speed regimes

**Fig. 3:**
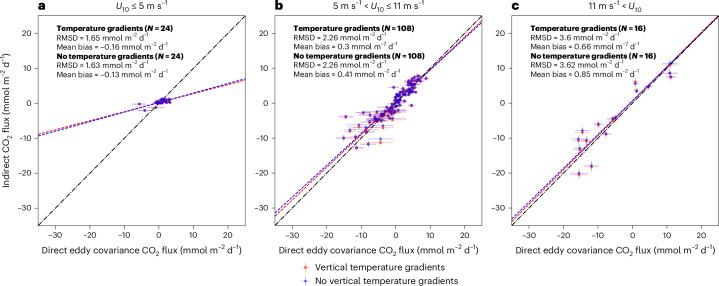
Scatter plots showing comparison between 3-h mean direct eddy covariance and indirect bulk CO_2_ fluxes grouped by average wind speeds. **a**, Comparison between 3-h mean direct eddy covariance and indirect bulk CO_2_ fluxes not accounting for and accounting for vertical temperature gradients using Donlon et al.^12^ cool skin for wind speeds less than 5 m s^−1^. Dashed lines indicate the Type II linear regression fit for the respective dataset. Dashed-dotted line is the 1:1. In-plot statistics are root mean square difference (RMSD), mean bias and number of 3 h mean samples (*N*). **b**, Same as **a** for wind speeds greater than 5 m s^−1^ and less than 11 m s^−1^. **c**, Same as **a** for wind speeds greater than 11 m s^−1^. Error bars in all panels indicate the calculated uncertainty on the 3 h mean.

Wind speed dependent gas transfer velocity (*K*) parameterizations have been used for indirect flux calculations for decades since high-quality global wind data are readily available^29,30^. These parameterizations are most robust at moderate wind speeds (5 to 11 m s^−1^) and can explain a substantial proportion of the variance in *K* under these conditions^28,31^. This is also apparent within our analysis, as the mean bias between direct and indirect CO_2_ fluxes at these wind speeds moves closest to 0.0 (from 0.23 to 0.12 mmol m^−2^ d^− 1^, *N* = 108; Fig. 3b).

For wind speeds greater than 11 m s^−1^, there was a larger absolute reduction in the bias between direct and indirect CO_2_ fluxes (from 0.46 to 0.26 mmol m^−2^ d^−1^) when including vertical temperature gradients (*N* = 16; Fig. 3c). The polar oceans, especially the Southern Ocean, can regularly experience high wind speeds and are therefore considered strong CO_2_ sinks^4,20^. Indeed, the physical oceanographic community have shown that the cool skin persists at these higher wind speeds^12^, whereas the warm layers are eroded (Supplementary Fig. 1). This indicates that accounting for the vertical temperature gradients within air–sea gas fluxes is still important at higher wind speeds. Our results also support the idea that accounting for vertical temperature gradients is especially important in polar regions, which is consistent with the results of Dong et al.^6^.

At wind speeds less than 5 m s^−1^, accounting for vertical temperature gradients, somewhat surprisingly, does not improve the agreement between direct and indirect flux (*N* = 24), possibly because *K* is less well constrained at these wind speeds (by these wind-speed-only-based parameterizations). Surfactants^32–34^, convectively driven turbulence^35,36^ and chemical enhancement^37^ are all considered to have an important influence on the surface turbulence and *K* at these low wind speeds, which will vary regionally. The presence of diurnal warming of surface waters alongside a cooler skin layer^13^ (Supplementary Figs. 1 and 4) promotes a more complex near-surface concentration gradient response at these low wind speeds than at higher wind speeds (where the cool skin is probably prevalent due to erosion of warm layers). Vertical temperature gradients, and the resultant bias, are therefore likely to be important at these low wind speeds, but their effect on the CO_2_ flux could be masked by the poorly constrained *K*.

Although our results are consistent with the theoretical change^9^ at moderate and high wind speeds, and support the case for increased ocean uptake by vertical temperature gradients, the improvement of including the temperature gradients for these in situ data are not, nor expected to be, statistically significant on their own (Mann Whitney *U* test, *P* = 0.70, *N* = 148). From the theory, the systematic bias change observed will be relatively small for each individual measurement and the magnitude of this bias will fall within the range of the random uncertainties. However, previous work has highlighted that the impact of this small bias becomes more significant once integrated across a larger dataset or region^4,6^. Therefore, to guide future efforts we assess the number of individual in situ observations required to reach significance by replicating our dataset (that is, increasing *N*) until significance is reached. This assessment shows a significance (*P* = 0.04) is reached when *N* = 3,700 3-h averages, which equates to continuous measurements for ~462 days (~1.25 years). In contrast, this work represents the results from 2 years of research cruises, which culminated in *N* = 148 measurements. So collecting continuous measurements for over a year (that is, to reach 3,700 measurements) would be a large, but important community undertaking that should seek to further confirm the impact of these gradients across all basins. These same data could also be used to evaluate novel or more constrained wind speed gas exchange parameterizations and could also form the beginning of the reference or fiducial dataset that is now needed for supporting global carbon observing capabilities^19^. Adapting these methods for use on a buoy could also provide a feasible route to collecting the large ~1.25 years’ worth of data.

## Atlantic-wide and global implications

A maximum reduction in the bias between the direct and indirect fluxes from 0.19 to 0.08 mmol m^−2^ d^−1^ was observed when accounting for natural vertical temperature gradients using the Donlon et al.^12^ cool skin (Table 1). Scaling the reduction in the bias to the Atlantic Ocean between 50° N and 50° S implies that the Atlantic Ocean CO_2_ sink should be 0.03 PgC yr^−1^ greater, which amounts to 7% for the recent Atlantic CO_2_ sink value of ~0.5 PgC yr^−1^ (ref. ^2^). This increase in the ocean CO_2_ sink is the result of two opposing bias corrections; the inclusion of the natural cool skin effect, which increases the sink and the correction of the CO_2_ fugacity at depth ($f_{{{\mathrm{CO}}_{2}}\,({\mathrm{sw}},{\mathrm{depth}})}$) to the subskin temperature (*T*_subskin_) to correct for natural warm layers, which generally reduces the sink (Fig. 1).

Using the in situ AMT dataset, we can separately evaluate the impacts of these two corrections (described in Methods). The cool skin correction results in a change in mean bias of 0.26 mmol m^−2^ d^−1^ (that is, +0.19 mmol m^−2^ d^−1^ to −0.07 mmol m^−2^ d^−1^; Table 1), which if scaled evenly across the global ocean area, is equivalent to a global CO_2_ sink change of ~−0.42 PgC yr^−1^ (negative indicates increased sink). This value is consistent with the equivalent estimates from observation-based global analyses which identified ~−0.4 PgC yr^−1^ (refs. ^4,6,9^) (Fig. 1).

Previous work suggested that the correction of $f_{{{\mathrm{CO}}_{2}}\,({\mathrm{sw}},{\mathrm{depth}})}$ to the *T*_subskin_ increased the global CO_2_ sink by ~−0.5 PgC yr^−1^ (ref. ^4^), whereas a recent observation-based analysis using an updated satellite *T*_subskin_ dataset revised this correction to ~−0.2 PgC yr^−1^ (Fig. 1)^6^. These corrections of $f_{{{\mathrm{CO}}_{2}}\,({\mathrm{sw}},{\mathrm{depth}})}$ to the *T*_subskin_ within this previous work^4,6^ are the combination of the natural variability between *T*_depth_ and *T*_subskin_ due to the presence of diurnal warm layers and an artificial component due to warming of samples both within ship seawater intakes themselves and within analytical instrumentation on the ship before the measurement is taken^7,38^ (Fig. 1). This artificial component is not present within the in situ AMT data presented here because the *T*_depth_ measurements were well calibrated (Methods). Consequently, for the two AMT cruises shown here, the change in uptake due to the bias correction of $f_{{{\mathrm{CO}}_{2}}\,({\mathrm{sw}},{\mathrm{depth}})}$ to the *T*_subskin_ (−0.07 mmol m^−2^ d^−1^ to +0.08 mmol m^−2^ d^−1^) is solely due to the natural warm layers. Scaling this change evenly across the global ocean area amounts to a reduced uptake of ~+0.24 PgC yr^−1^, which should not be directly compared to the previous observation-based estimates^4,6^ due to the omission of the artificial component (Fig. 1).

Overall, accounting for the combination of the global ocean CO_2_ sink increase by the natural cool skin and the opposing reduction by the naturally occurring warm layers would (based on two scaling calculations; Methods) suggest an ~0.18 PgC yr^−1^ increase in the global CO_2_ sink. Using different cool skin parameterizations does not alter the sign but does influence the magnitude of this adjustment in the ocean sink (Supplementary Table 4). Bellenger et al.^39^ indicated an increase in the net integrated global ocean sink of 0.13 PgC yr^−1^ within a global Earth System Model when natural vertical temperature gradients and the salty skin were accounted for. The work here has not included the salty skin (due to constraints in collecting relevant measurements), but Woolf et al.^9^ suggest the salty skin reduces global net CO_2_ uptake by ~0.05 PgC yr^−1^. The results in this paper are therefore consistent with Bellenger et al.^39^ (that is, 0.18 PgC yr^−1^ from our results minus 0.05 = 0.13 PgC yr^−1^; Fig. 1). Neither study includes any near-surface chemical effects, but the impact of these effects on the air–sea CO_2_ flux is a topic of discussion (for example, see the differing views within refs. ^9,39,40^). A large in situ dataset following the approach presented here could advance understanding on this issue. Overall, the 0.18 PgC yr^−1^ bias due to neglecting natural vertical temperature gradients equates to a 6% underestimation of the global ocean sink (based upon a global sink of 2.9 PgC yr^−1^) (ref. ^2^), which agrees with the theory^9^, previous observation-based global assessments^3,4,6^ and recent modelling study advances^39^.

## Conclusion

In this study, a comprehensive dataset of in situ direct eddy covariance and indirect estimates of air–sea CO_2_ fluxes with high accuracy and well-characterized uncertainties were collected along two transects in the Atlantic Ocean. The measurements included temperatures at depth (*T*_depth_) and over the ocean’s skin (*T*_skin_), which allowed for the surface vertical temperature gradients to be characterized. These data enable a targeted large-scale field evaluation of the effects of natural vertical temperature gradients on air–sea CO_2_ fluxes. Explicitly considering the vertical temperature gradients in the indirect CO_2_ flux calculation improved the agreement with the direct eddy covariance fluxes. The mean difference between the indirect and direct CO_2_ fluxes was reduced from 0.19 to 0.08 mmol m^−2^ d^−1^ and when scaled to the Atlantic Ocean (50° N to 50° S) indicated an upward adjustment in the CO_2_ sink of ~0.03 PgC yr^−1^ or 7% of the Atlantic ocean’s CO_2_ sink.

When extrapolated evenly to the global ocean area, the results imply an ~0.42 PgC yr^−1^ increase in the global ocean CO_2_ sink due to the cool skin and an opposing ~0.24 PgC yr^−1^ decrease due to natural warm layers. This work provides in situ observational evidence that the bias error caused by ignoring vertical temperature gradients should be considered when calculating air–sea CO_2_ fluxes from bulk approaches within global carbon assessments. The inclusion of vertical temperature gradients has reduced the bias within the indirect fluxes, which in turn has increased the accuracy of the global ocean CO_2_ sink estimates, whereas the precision of the ocean CO_2_ sink estimates has remained the same. These results agree with the theory, previous global observation-based studies and a recent modelling study. The results highlight the need for the continued collection of high-quality data to further verify these signals across all ocean basins.

## Methods

The 28th (September–October 2018) and 29th (October–November 2019) AMT research cruises (AMT28 and AMT29, respectively) traversed the Atlantic Ocean from north to south, including the remote North and South Atlantic gyres (Fig. 2a). Data were available for the whole cruise track on AMT29 (~50° N to 50° S), but on AMT28, due to instrumentation issues, data were only available for the South Atlantic (~5° N to 50° S).

### In situ measurements and calculations for the direct eddy covariance air–sea CO_2_ flux estimates

The eddy covariance technique provides consistent and reliable direct measurements of the CO_2_ flux (Flux_EC_) from ships in unprecedented detail and at high frequency^27,41–44^. Direct CO_2_ flux measurements are made purely in the atmosphere and do not require any seawater data. In the case of CO_2_, this micro-meteorological technique combines high frequency (10 Hz) measurements of vertical wind velocity (*w*) and the dry mixing ratio of CO_2_ in the atmosphere ($x_{{\mathrm{CO}}_{2}\,({\mathrm{atm}})}$) from the foremast of the ship to derive the net vertical CO_2_ flux. During AMT28, a cavity ringdown analyser (Picarro G2311-f) was used, whereas an infrared absorption analyser (Li-Cor 7200) was used during AMT29^45^. Both systems were dried with a Nafion dryer to remove the effect of water vapour on CO_2_ flux. Wind and ship motion data were measured with a 3D sonic anemometer (Metek uSonic-3 during AMT28, Gill R3-50 during AMT29) and an inertial measurement unit (Systron Donner Motionpak II during AMT28, LPMS during AMT29). The wind data were then motion-corrected following Edson et al.^46^ and Dong et al.^45^. The CO_2_ flux in mixing ratio units (ppm m sec^−1^) was converted to molar concentration units (mmol m^−2^ d^−1^) using the mean dry air density (*ρ*_dry_), derived from measurements of air temperature, humidity and pressure, following:

1 $${\mathrm{Flux}}_{\mathrm{EC}}={\rho }_{\mathrm{dry}}\,\overline{{w}^{{\prime} }{X}_{{\mathrm{CO}}_{2}}^{{\prime} }}$$

Here the overbar indicates a 20-min mean, which was the initial averaging interval. The 20-min fluxes are quality controlled to remove unfavourable measurement periods and corrected for high frequency flux attenuation. The measurement uncertainties are calculated following Dong et al.^45^. Detailed descriptions of the eddy covariance set-up and quality control procedures for both cruises are provided in Dong et al.^45^.

The 20-min direct CO_2_ fluxes are accurate observations but individually have a low precision due to a relatively large random noise component^45^. The random noise can be reduced by averaging 20-min fluxes over longer time periods to increase the signal-to-noise ratio. Dong et al.^45^ showed that for regions of low CO_2_ fluxes, the averaging time to reach a 3:1 signal-to-noise ratio is about 3 h. Yang et al.^27^ also showed that for regions with a near equilibrium CO_2_ concentration gradient, the direct CO_2_ fluxes showed no significant bias. Therefore, by averaging the 20-min direct CO_2_ fluxes over 3 h, these observations are accurate and have no discernible bias, with a total absolute uncertainty in the order of 1 mmol m^−2^ d^−1^ (relative uncertainty of ~31%)

### In situ measurements for indirect bulk air–sea CO_2_ flux estimates

Environmental parameters were measured concurrently for the indirect bulk estimation of air–sea CO_2_ fluxes using the ships’ underway system. The underway system collected continuous along-track measurements of inherent oceanographic properties by drawing water into the vessels through an inlet at ~6 m below the sea surface. Sea temperature and salinity at this depth were measured with sensors at the inlet pipe, and these data were returned to the British Oceanographic Data Centre (BODC) for initial calibration, quality control and processing into 1-s averages. These *T*_depth_ measurements were further calibrated against coincident measurements using an external temperature sensor on discrete conductivity, temperature and depth sensor casts along both cruise tracks (Supplementary Fig. 2; AMT28 *N* = 29, AMT29 *N* = 49).

On AMT28, measurements of $f_{{\mathrm{CO}}_{2}\,({\mathrm{sw}},{\mathrm{depth}})}$ were made from the same water intake as the *T*_depth_ and salinity measurements using the PML-Dartcom Live-pCO_2_ system^47^. This system was calibrated hourly using secondary CO_2_ standards (BOC Gases Ltd.; nominal 250, 380 and 450 ppmv CO_2_ in synthetic air), which were themselves calibrated against reference standards from the National Oceanic and Atmospheric Administration (244.9 and 444.4 ppmv CO_2_). Quality control of $f_{{\mathrm{CO}}_{2}\,({\mathrm{sw}},{\mathrm{depth}})}$ data followed standard best practices^48^. The $x_{{\mathrm{CO}}_{2}\,({\mathrm{atm}})}$ was measured from the front of the bridge ( ~16 m above water) using the same $f_{{\mathrm{CO}}_{2}}$ system and calibrated in the same fashion.

On AMT29, due to instrumentation failure with the PML-Dartcom Live-pCO_2_ system, a Segmented Flow Coil Equilibrator (SFCE) system^34,49^) measured $f_{{\mathrm{CO}}_{2}\,({\mathrm{sw}},{\mathrm{depth}})}$ and $x_{{\mathrm{CO}}_{2}\,({\mathrm{atm}})}$. Yang et al.^34^ performed a comparison between the SFCE and PML-Dartcom Live-pCO_2_ systems during a Southern Ocean cruise, showing good agreement (mean bias = 1.63 µatm; RMSD = 4.39 µatm) between the systems. A comparison during AMT29 between SFCE $f_{{\mathrm{CO}}_{2}\,({\mathrm{sw}},{\mathrm{depth}})}$ and $f_{{\mathrm{CO}}_{2}\,({\mathrm{sw}},{\mathrm{depth}})}$ estimated from dissolved inorganic carbon and total alkalinity discrete measurements using CO2SYSv3^50–53^ also showed good agreement (Supplementary Fig. 3; mean bias = 1.92 µatm, RMSD = 8.2 µatm, *N* = 13).

*T*_skin_ measurements were made using an Infrared Sea Surface Temperature Autonomous Radiometer (ISAR)^54,55^. On AMT28 the ISAR was mounted on the port side of the forward mast at a 45° angle relative to the centre line of the ship, and on AMT29 the angle relative to the centre line of the ship was 90°. Data were logged at 2-s intervals. Although the ISAR is a self-calibrating radiometer, to enable data to be true reference measurements, the instrument was calibrated before and after deployments.

Wind speed measured by the eddy covariance system was adjusted to 10 m neutral wind speed (*U*_10n_) using the COARE 3.5 model^18^. Air pressure (*P*), relative humidity (RH) and air temperature (*T*_air_) were measured using the ship meteorological sensor package. All in situ observations were measured at their native temporal resolution and averaged (mean) to 20-min windows, coincident to the eddy covariance flux observations (lowest common time denominator).

Subskin temperature (*T*_subskin_) was computed from *T*_skin_ using three estimates of the cool skin effect: (1) assumed to be a fixed 0.17 K (ref. ^11^); (2) calculated using the empirical wind speed relationship described in Donlon et al.^12^; (3) calculated with the COARE 3.5^13,18,56^ using in situ observations (*U*_10n_, *P*, *T*_skin_, RH, *T*_air_). For the COARE 3.5 model, in situ observations were not available for the incoming shortwave and longwave radiation, and therefore ERA5 estimates (hourly at 0.25° spatial resolution)^57^ were used for the nearest hour with a weighted mean based on spatial distance to the four closest observations. Supplementary Fig. 4 shows the differences in the cool skin estimates. The Donlon et al.^12^ and COARE 3.5 showed similar cool skin effects, except in periods where *T*_air_ was greater than the *T*_skin_ suggesting sensible heat gain to the ocean’s surface. But we note that the COARE 3.5 approach required model re-analysis data for incoming and outgoing radiation, and we have no way of confirming these data for the individual cruise dates.

### Indirect bulk air–sea CO_2_ flux calculations

Indirect bulk CO_2_ fluxes were computed using the FluxEngine toolbox (version 4.0.7)^58,59^. The Python toolbox enables user-configurable calculations that can be run with any combination of data from in situ, Earth observation and models for consistent bulk air–sea flux calculations. The toolbox scripts were configured to run using in situ observations described above at a temporal resolution of 20 min.

Indirect bulk fluxes (Flux_Bulk_) were firstly calculated assuming no vertical temperature gradients (the standard approach within the ocean carbon community over the last decades), using *T*_depth_ as the temperature for all components of the calculations;

2 $${\mathrm{Flux}}_{\mathrm{Bulk}}\approx {K\alpha }_{\mathrm{depth}}(\;f_{{\mathrm{CO}}_{2}\,({\mathrm{sw}},{\mathrm{depth}})}-f_{{\mathrm{CO}}_{2}\,({\mathrm{atm}})})$$

where *α*_depth_ is the solubility of CO_2_ in seawater at *T*_depth_ calculated following Weiss^60^. The gas transfer velocity, *K*, was computed using the gas transfer parameterization of Wanninkhof^28^ as the central estimate of commonly used gas transfer parameterizations^25–27^. But the calculations were also repeated for all other commonly used gas transfer parameterizations. $f_{{\mathrm{CO}}_{2}\,({\mathrm{atm}})}$ was calculated from $x_{{\mathrm{CO}}_{2}\,({\mathrm{atm}})}$, *T*_depth_, salinity and air pressure following Dickson et al.^48^.

Indirect bulk flux calculations were then repeated accounting for vertical temperature gradients. Initially we approximate the effect of the cool skin as:

3 $${\mathrm{Flux}}_{\mathrm{Bulk}}=K\Delta C\approx K\left({\alpha }_{\mathrm{depth}}\;f_{{\mathrm{CO}}_{2}\,({\mathrm{sw}},{\mathrm{depth}})}-{\alpha }_{\mathrm{skin}}\;f_{{\mathrm{CO}}_{2}\,({\mathrm{atm}})}\right)$$

where *α*_skin_ and $f_{{\mathrm{CO}}_{2}\,({\mathrm{atm}})}$ were recalculated using *T*_skin_ estimated from *T*_depth_ (using the three cool skin estimates; *T*_skin_ ≈ *T*_depth_ – cool skin).

Finally, to fully account for vertical temperature gradients, we use the in situ *T*_skin_ measurement and include the correction of $f_{{\mathrm{CO}}_{2}\,({\mathrm{sw}},{\mathrm{depth}})}$ to the *T*_subskin_:

4 $${\mathrm{Flux}}_{\mathrm{Bulk}}=K\Delta C=K\left({\alpha }_{\mathrm{subskin}}\;f_{{\mathrm{CO}}_{2}\,({\mathrm{sw}},{\mathrm{subskin}})}-{\alpha }_{\mathrm{skin}}\;f_{{\mathrm{CO}}_{2}\,({\mathrm{atm}})}\right)$$

where *α*_subskin_ was calculated using *T*_subskin_. *α*_skin_ and $f_{{\mathrm{CO}}_{2}\,({\mathrm{atm}})}$ were recalculated using the ISAR *T*_skin_. $f_{{\mathrm{CO}}_{2}\,({\mathrm{sw}},{\mathrm{depth}})}$ was corrected for carbonate equilibrium from *T*_depth_ to *T*_subskin_ (computed using the three cool skin estimates from the ISAR *T*_skin_; *T*_subskin_ = *T*_skin_ + cool skin) following Takahashi et al.^61^, with updated coefficients in Wanninkhof et al.^62^:

5 $${{f}_{{\mathrm{CO}}_{2}({\mathrm{sw}},{\mathrm{subskin}})}}={f_{{\mathrm{CO}}_{2}\,({\mathrm{sw}},{\mathrm{depth}})}\times\mathrm{e}}^{0.0413\left({T}_{\mathrm{subskin}}-{T}_{\mathrm{depth}}\right)}$$

Uncertainties within the input parameters were propagated through the indirect bulk CO_2_ flux calculations using a Monte Carlo uncertainty propagation with 100 ensembles. The standard deviation of the distribution from which the random noise values were drawn was as follows. For *U*_10n_ (m s^−1^) the measurement uncertainty was calculated as ±3% of each value in the dataset. This included uncertainty in the sonic anemometer wind measurement and the uncertainty due to potential wind distortion around the ship superstructure. An uncertainty of ±4 μatm was applied to underway $f_{{\mathrm{CO}}_{2}\,({\mathrm{sw}},{\mathrm{depth}})}$ measurements, following results in Ribas-Ribas et al.^63^ and comparisons in Yang et al.^34^. On the basis of the calibration of the systems, an uncertainty of ±1 ppm was applied to the $x_{{\mathrm{CO}}_{2}\,({\mathrm{atm}})}$ dataset. A variable *T*_skin_ uncertainty was determined using the ISAR uncertainty model (v4.5)^55^. An uncertainty of 0.1 K was applied to *T*_depth_.

The indirect flux measurement uncertainty was extracted as two standard deviations (95% confidence interval) of the ensembles. Assuming that these uncertainties are uncorrelated, this was combined in quadrature^64^ with the estimated uncertainties related to *K* of 10% (ref. ^10^). This corresponds to a mean absolute indirect bulk flux uncertainty of 1.4 mmol m^−2^ d^−1^ (mean relative uncertainty of ~35%).

### Statistical comparison of direct eddy covariance and indirect bulk CO_2_ fluxes

Direct and indirect CO_2_ fluxes were compared and contrasted using mean bias (indirect bulk flux – direct eddy covariance flux), root mean square difference (RMSD), slope and intercept of Type II reduced major axis linear regression and the number of measurements (*N*). The slope and intercept of a Type II regression were computed because uncertainties are present in both the direct and indirect CO_2_ fluxes. Due to the lack of any discernible bias in the eddy covariance flux data^45^, the calculated mean bias is attributed to the indirect bulk fluxes.

The direct and indirect fluxes were compared using 3-h static window averages (mean) where at least two-thirds of the data for the window were available. The 3-h time window increases the signal-to-noise ratio of the direct fluxes as recommended by Dong et al.^45^. Here both the indirect and direct measurements and their uncertainties were averaged to a consistent and aligned temporal window. The 20-min measurement uncertainties were propagated within the 3-h time window as^64^

6 $${\mathrm{unc}}_{(\mathrm{meas},3\,\mathrm{h})}=\frac{\sqrt{\sum ({{\mathrm{unc}}_{\left(\mathrm{meas},20\,{\rm{min}}\right)}}^{2})}}{n}$$

### Scaling bias reductions globally

The global ocean area was calculated for a 1° latitude and longitude grid assuming Earth is an ellipsoid and a higher spatial resolution land percentage mask supplied within FluxEngine was applied. The total area for the Atlantic Ocean was calculated using the supplied Atlantic Ocean mask within FluxEngine and a latitude band between 50° N and 50° S. The change in mean bias between direct and indirect CO_2_ fluxes when accounting and not accounting for vertical temperature gradients (mmol m^−2 ^d^−1^) was multiplied by the Atlantic Ocean area (km^2^) and converted to PgC yr^−1^ equivalents. The calculation was then repeated for the global ocean area estimated from the same 1° grid and land mask.

A second global scaling approach was applied to the wind speed dependent mean bias changes due to vertical temperature gradients (Fig. 3). The mean bias change (mmol m^−2^ d^−1^) for each of the wind speed bands (Fig. 3) were applied to 1° mean monthly cross-calibrated wind speeds (CCMP) v3.1^30,65^ from 2018 and multiplied by the area and land percentage mask used previously. These values were integrated globally and converted to PgC yr^−1^. This scaling approach yielded consistent results (that is, within three decimal places on the integrated value) to the previous fixed global scaling approach.

## Online content

Any methods, additional references, Nature Portfolio reporting summaries, source data, extended data, supplementary information, acknowledgements, peer review information; details of author contributions and competing interests; and statements of data and code availability are available at 10.1038/s41561-024-01570-7.

## Supplementary information

Supplementary Information Supplementary Figs. 1–4 and Tables 1–4.

## Data Availability

The 20-min averaged (mean) in situ observations are available via Zenodo at 10.5281/zenodo.13691315 (ref. ^66^).

## Code availability

The analysis code is available via Github at https://github.com/JamieLab/AMT4CO2FLUX and with the in situ observations via Zenodo at 10.5281/zenodo.13691315 (ref. ^66^).
