# Supplementary material for: Enhanced ocean CO2 uptake due to near-surface temperature gradients
Source: Nat Geosci. 2024 Oct 25;17(11):1135–40. doi: 10.1038/s41561-024-01570-7 (PMC12321578; doi:10.1038/s41561-024-01570-7)
Supplement: Supplementary file 1 — Supplementary Figs. 1–4 and Tables 1–4. [file 41561_2024_1570_MOESM1_ESM.pdf]

nature geoscience

Supplementary information https://doi.org/10.1038/s41561-024-01570-7

Enhanced ocean CO_2 uptake due to near-surface temperature gradients

In the format provided by the authors and unedited

Table of Contents

- Table S1 (Page 2)
- Table S2 (Page 3)
- Table S3 (Page 4)
- Table S4 (Page 4)
- Figure S1 (Page 5)
- Figure S2 (Page 6)
- Figure S3 (Page 7)
- Figure S4 (Page 8)
- References (Page 9)

Version 2 of the Supplementary Information

Tables S1, S2 and S3 have been updated in an Author Correction. All remaining Figures and Tables are unchanged

Table S1: Statistical comparisons between direct and indirect CO_2 fluxes not accounting for vertical temperature gradients (VTG) and accounting for VTG using different cool skin estimates and the Ho et al. [1] gas transfer parameterisation. Statistical acronyms are mean bias, root mean square difference (RMSD) and number of observations (N). **Values in square brackets indicate the values in the original published version.**

<table>
<tr><th colspan="2">Method</th><th>Mean Bias
(mmol m^{-2} d^{-1})</th><th>RMSD
(mmol m^{-2} d^{-1})</th><th>Slope</th><th>Intercept</th><th>N</th></tr>
<tr><td colspan="2">No VTG
(Eq. 2)</td><td>0.37 [0.19]</td><td>2.36 [2.34]</td><td>0.93 [0.94]</td><td>0.33 [0.15]</td><td>148</td></tr>
<tr><td rowspan="3">Cool Skin Correction
(Eq. 3)</td><td>Fixed Skin (0.17K)</td><td>0.11 [-0.08]</td><td>2.33 [2.33]</td><td>0.93 [0.94]</td><td>0.07 [-0.11]</td><td>148</td></tr>
<tr><td>Donlon et al. [2] Skin</td><td>0.11 [-0.07]</td><td>2.33 [2.33]</td><td>0.93 [0.94]</td><td>0.07 [-0.10]</td><td>148</td></tr>
<tr><td>COARE Skin</td><td>0.04 [-0.14]</td><td>2.34 [2.34]</td><td>0.93 [0.94]</td><td>0.01 [-0.17]</td><td>148</td></tr>
<tr><td rowspan="3">Cool Skin and
Warm layer correction
(Eq. 4)</td><td>Fixed Skin (0.17K)</td><td>0.26 [0.08]</td><td>2.37 [2.35]</td><td>0.95 [0.96]</td><td>0.23 [0.06]</td><td>148</td></tr>
<tr><td>Donlon et al. [2] Skin</td><td>0.26 [0.08]</td><td>2.37 [2.35]</td><td>0.95 [0.96]</td><td>0.23 [0.06]</td><td>148</td></tr>
<tr><td>COARE Skin</td><td>0.29 [0.11]</td><td>2.37 [2.35]</td><td>0.95 [0.96]</td><td>0.27 [0.09]</td><td>148</td></tr>
</table>

Table S2: Statistical comparisons between direct and indirect CO_2 fluxes not accounting for vertical temperature gradients (VTG) and accounting for VTG using different cool skin estimates and the Nightingale et al. [3] gas transfer parameterisation. Statistical acronyms are mean bias, root mean square difference (RMSD) and number of observations (N). **Values in square brackets indicate the values in the original published version.**

Method		Mean Bias (mmol m^{-2} d^{-1})	RMSD (mmol m^{-2} d^{-1})	Slope	Intercept	N
No VTG (Eq. 2)		0.41 [0.24]	2.36 [2.33]	0.88 [0.89]	0.35 [0.18]	148
Cool Skin Correction (Eq. 3)	Fixed Skin (0.17K)	0.16 [-0.02]	2.32 [2.31]	0.90 [0.90]	0.10 [-0.07]	148
	Donlon et al. [2] Skin	0.16 [-0.02]	2.32 [2.31]	0.90 [0.90]	0.10 [-0.07]	148
	COARE Skin	0.10 [-0.08]	2.32 [2.32]	0.90 [0.90]	0.04 [-0.14]	148
Cool Skin and Warm layer correction (Eq. 4)	Fixed Skin (0.17K)	0.31 [0.13]	2.35 [2.33]	0.91 [0.91]	0.26 [0.09]	148
	Donlon et al. [2] Skin	0.31 [0.13]	2.35 [2.33]	0.91 [0.91]	0.26 [0.09]	148
	COARE Skin	0.34 [0.17]	2.35 [2.33]	0.91 [0.91]	0.29 [0.12]	148

Table S3: Statistical comparisons between direct and indirect CO_2 fluxes not accounting for vertical temperature gradients (VTG) and accounting for VTG using different cool skin estimates and the Yang et al. [4] gas transfer parameterisation. Statistical acronyms are mean bias, root mean square difference (RMSD) and number of observations (N). **Values in square brackets indicate the values in the original published version.**

Method		Mean Bias (mmol m^{-2} d^{-1})	RMSD (mmol m^{-2} d^{-1})	Slope	Intercept	N
No VTG (Eq. 2)		0.50 [0.28]	2.56 [2.54]	1.08 [1.09]	0.54 [0.33]	148
Cool Skin Correction (Eq. 3)	Fixed Skin (0.17K)	0.18 [-0.04]	2.52 [2.53]	1.09 [1.10]	0.23 [0.01]	148
	Donlon et al. [2] Skin	0.18 [-0.04]	2.52 [2.53]	1.09 [1.10]	0.23 [0.01]	148
	COARE Skin	0.10 [-0.12]	2.53 [2.54]	1.09 [1.09]	0.15 [-0.07]	148
Cool Skin and Warm layer correction (Eq. 4)	Fixed Skin (0.17K)	0.37 [0.15]	2.60 [2.59]	1.11 [1.12]	0.43 [0.21]	148
	Donlon et al. [2] Skin	0.37 [0.15]	2.60 [2.59]	1.11 [1.12]	0.43 [0.21]	148
	COARE Skin	0.42 [0.20]	2.60 [2.59]	1.11 [1.12]	0.47 [0.26]	148

Table S4: Global extrapolation of the bias difference between direct eddy covariance and indirect bulk CO_2 fluxes for different cool skin parameterisations. The global correction due to the cool skin, warm layers, and the net global effect of the two corrections are calculated. Negative values indicate increased ocean CO_2 uptake.

Cool skin parameterisation	Cool skin effect (Pg C yr^{-1})	Warm Layer effect (Pg C yr^{-1})	Net effect (Pg C yr^{-1})
Fixed Skin (0.17K)	-0.42	+0.26	-0.16
Donlon et al. [2] Skin	-0.42	+0.24	-0.18
COARE Skin	-0.51	+0.40	-0.11

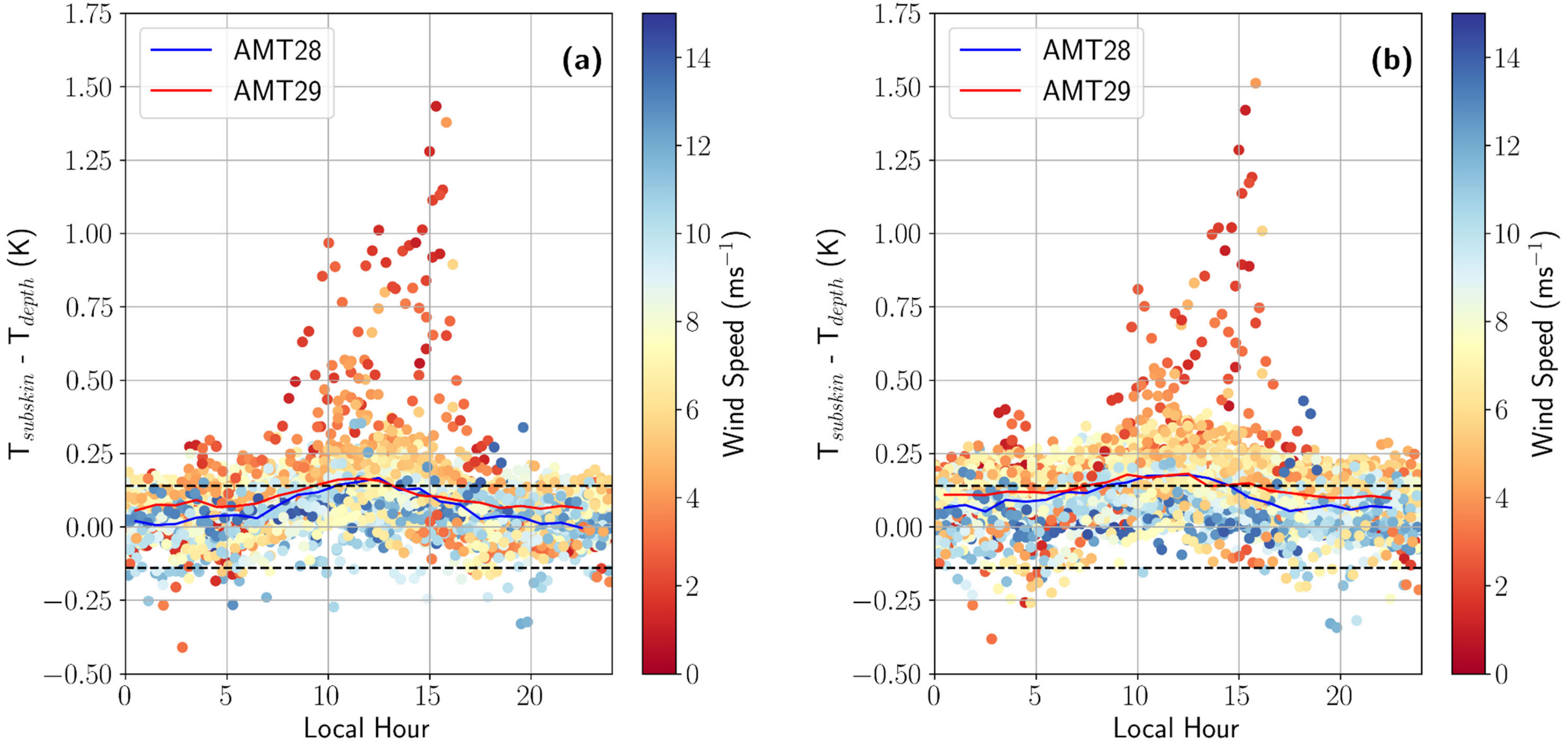

Figure S1: The difference between subskin ($T_{subskin}$; ~2 mm) and depth (T_{depth}; ~6 m) temperatures over 24-hour periods indicating the magnitude of the warm layers and the wind conditions under which they formed on AMT28 and AMT29 (a) 20-minute $T_{subskin}$ - T_{depth} using the Donlon et al. [2] cool skin to estimate $T_{subskin}$ from T_{skin} plotted against local time. Colour indicates the wind speed (ms^{-1}). Solid lines indicate median temperature difference. Dashed lines indicate an average uncertainty window for $T_{subskin} – T_{depth}$ of ~±0.14K. (b) same as (a) but using the NOAA COARE cool skin.

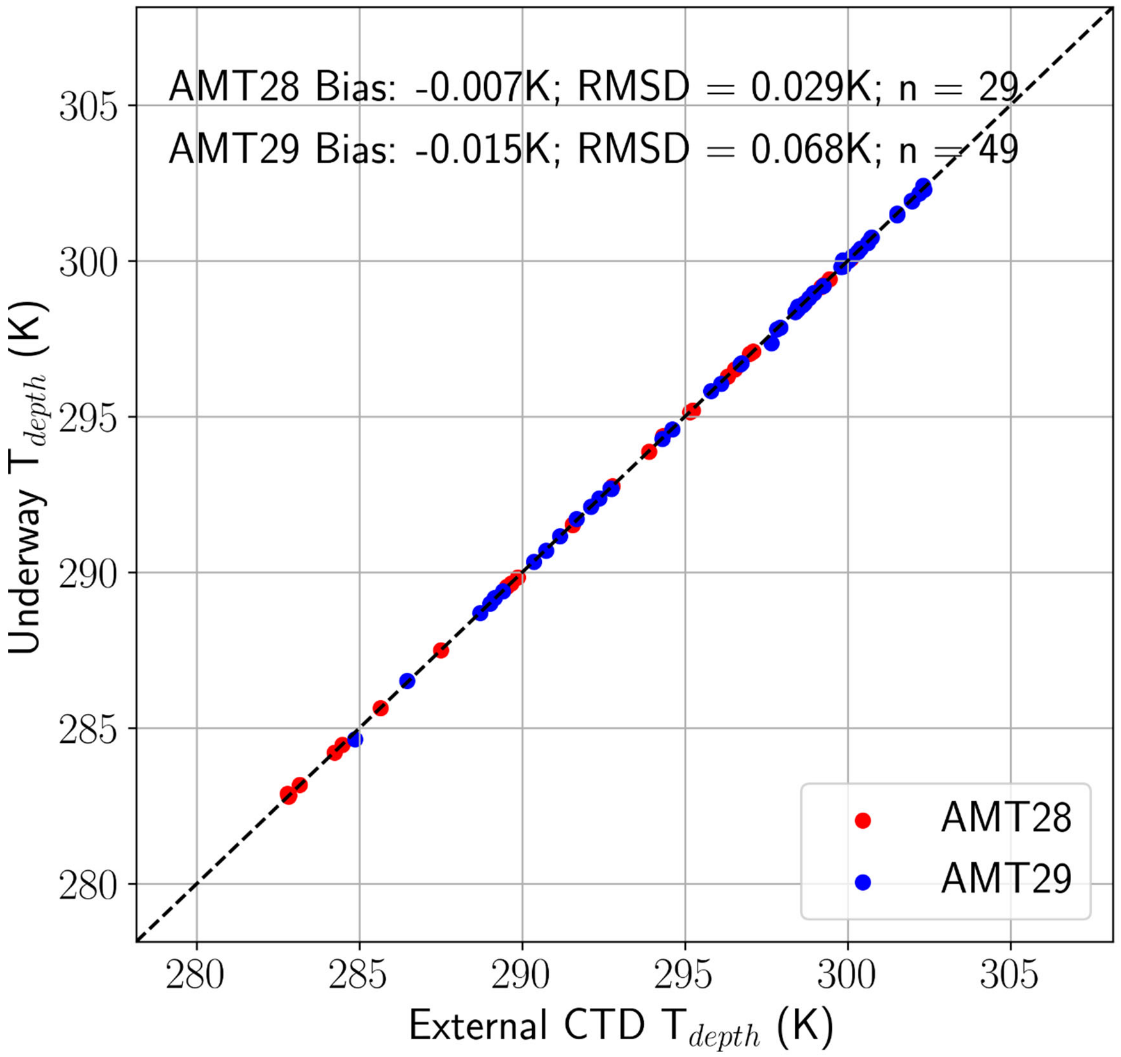

Figure S2: Underway temperature at depth (T_{depth}; ~6 m) was corrected to an external T_{depth} sensor with a residual bias of 0.007 K on AMT28 and 0.015 K on AMT29. Figure shows a comparison between underway T_{depth} from a sensor within the research vessel and an external T_{depth} from discrete conductivity, temperature, and depth (CTD) sensor deployments. Dashed line is the 1 to 1 line. In text statistics are bias, root mean square deviation (RMSD) and number of matchups between underway and external T_{depth} sensor (n).

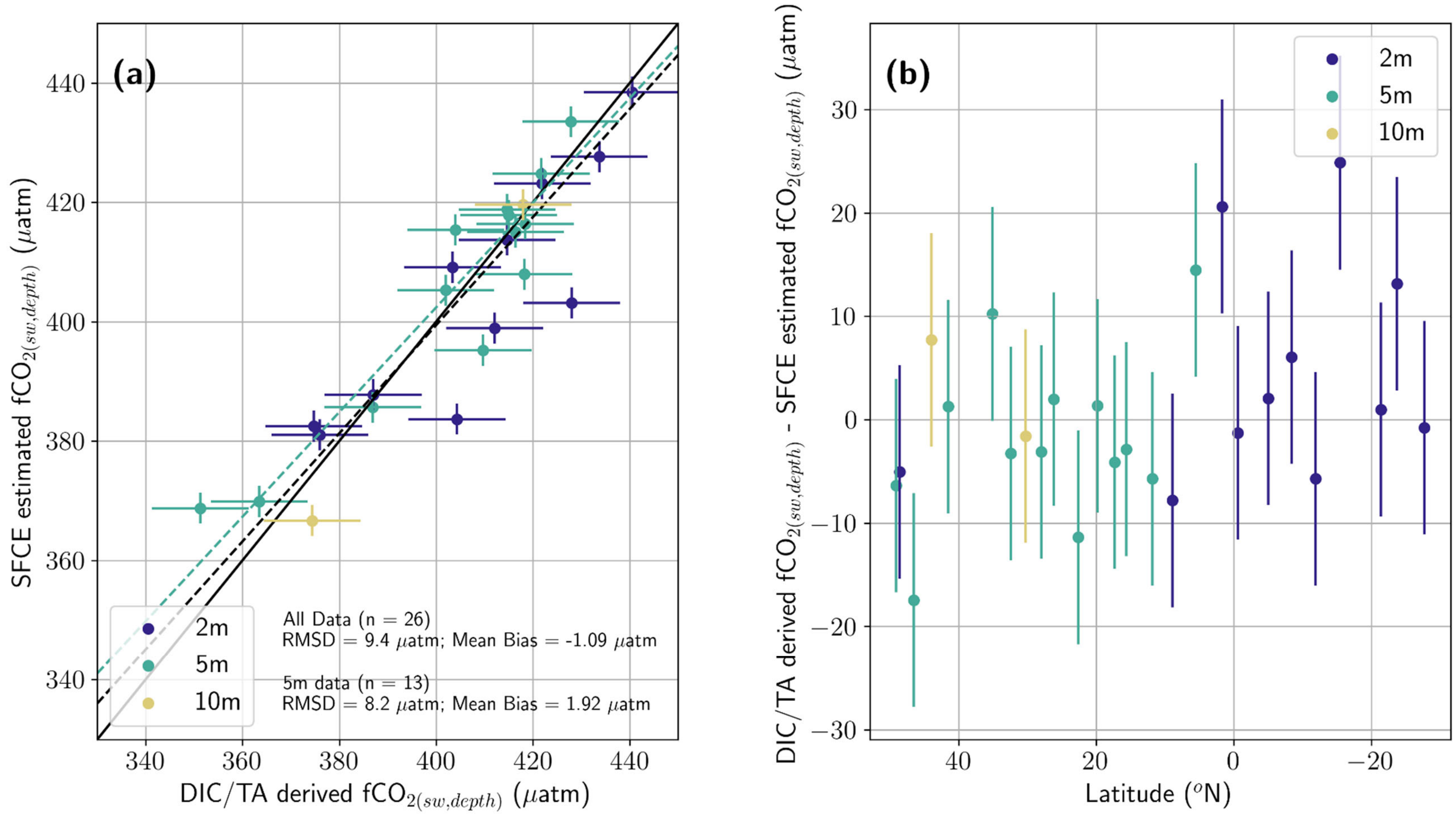

Figure S3: A Segmented Flow Coil Equilibrator (SFCE) was used on AMT29 to measure $fCO_{2\ (sw,depth)}$, which was compared to $fCO_{2\ (sw,depth)}$ derived from dissolved inorganic carbon (DIC) and total alkalinity (TA), which agreed well with a mean bias of -1.9 µatm, and a root mean square difference (RMSD) of 8.2 µatm. This RMSD is within the ~±10 µatm uncertainty of the $fCO_{2\ (sw,depth)}$ from DIC and TA. (a) Comparison between $fCO_{2\ (sw,depth)}$ derived from discrete DIC and TA measurements within the top 10 m and SFCE $fCO_{2\ (sw,depth)}$ from ~6 m depth. Dashed line indicates the type-II linear regression fit for 5m data (coloured) and all data (black). Solid line is the 1:1. In text stats are root mean square difference (RMSD), mean bias and number of samples (n). Errorbars indicate the calculated uncertainty on the SFCE (y-axis) and calculated uncertainty on the $fCO_{2\ (sw)}$ from DIC and TA (x-axis). (b) Difference between $fCO_{2\ (sw,depth)}$ derived from discrete DIC and TA measurements and SFCE $fCO_{2\ (sw,depth)}$ plotted against latitude. Errorbars indicate the combined calculated uncertainties on the SFCE and $fCO_{2\ (sw)}$ from DIC and TA.

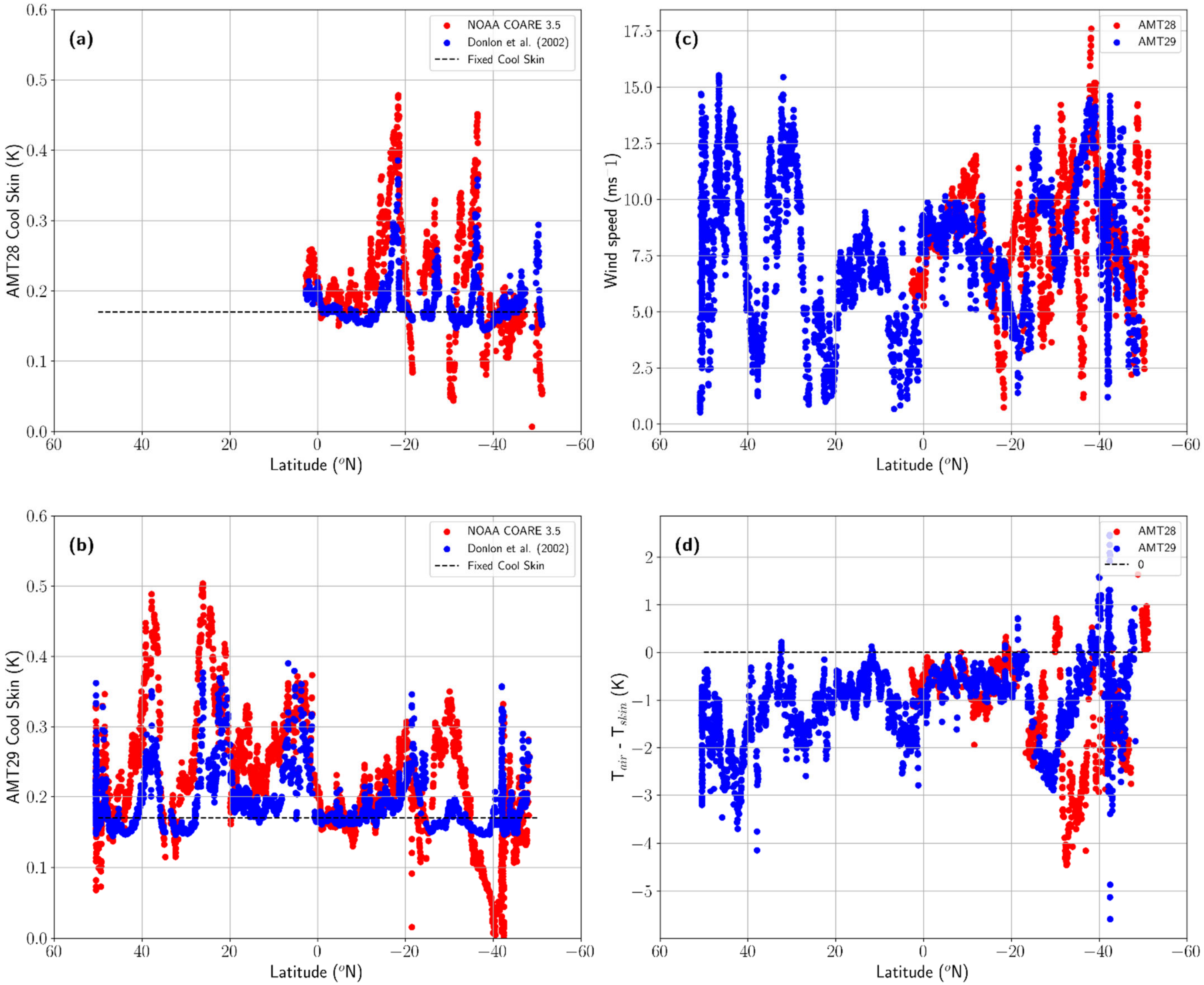

Figure S4: (a) Cool skin on AMT28 plotted against latitude. (b) same as (a) for AMT29. (c) Wind speed for AMT28 and AMT29 plotted against latitude. (d) Air temperature (T_{air}) minus sea surface skin temperature (T_{skin}) for AMT28 and AMT29 plotted against latitude. Dashed line indicates 0.

References

1. Ho, D. T. *et al.* Measurements of air-sea gas exchange at high wind speeds in the Southern Ocean: Implications for global parameterizations. *Geophysical Research Letters* **33**, L16611 (2006).

2. Donlon, C. J. *et al.* Toward Improved Validation of Satellite Sea Surface Skin Temperature Measurements for Climate Research. *Journal of Climate* **15**, 353–369 (2002).

3. Nightingale, P. D. *et al.* In situ evaluation of air-sea gas exchange parameterizations using novel conservative and volatile tracers. *Global Biogeochemical Cycles* **14**, 373–387 (2000).

4. Yang, M. *et al.* Global Synthesis of Air-Sea CO_2 Transfer Velocity Estimates From Ship-Based Eddy Covariance Measurements. *Frontiers in Marine Science* **9**, 826421 (2022).

5. Donlon, C. J. *et al.* Implications of the oceanic thermal skin temperature deviation at high wind speed. *Geophysical Research Letters* **26**, 2505–2508 (1999).

6. Edson, J. B. *et al.* On the Exchange of Momentum over the Open Ocean. *Journal of Physical Oceanography* **43**, 1589–1610 (2013).
